# Supplementary material for: Is massage a legitimate part of nursing care? A qualitative study
Source: PLoS One. 2023 Feb 27;18(2):e0281078. doi: 10.1371/journal.pone.0281078 (PMC9970055; doi:10.1371/journal.pone.0281078)
Supplement: Attachment — Submitted filename: Comment Reviewer.pdf [file pone.0281078.s001.pdf]

Is massage a legitimate part of nursing care? A qualitative study

Comments to the Author

Congratulation on the submitted manuscript. The topic is timely and will be of interest to the readers of the journal. However, few changes are suggested to improve the clarity of this manuscript.
I have several recommendations and questions about the manuscript.

No	Page, Para	Descriptions of item	Comment
1.		Abstract	A qualitative study approach was used for this study involving how many participants? It is necessary to provide the total number of participants in your approach at the beginning of your statement.<br>The inclusion criteria for the study need to be discussed here.<br>List the Five themes that emerged from the thematic analysis in the abstract.
2	4,70	Introduction	Need to explain the full meaning of The ICD-11 for the first use in a sentence.
3	 <br>6,134<br><br><br><br><br><br>6,137<br><br><br><br><br><br><br>6,139	Methodology<br><br>An intervention (TM or control) was assigned to each of the two units/clusters.<br><br><br>The HCPs received training on TM or the use of the machine according to their unit (CG or IG).<br><br><br>Ethical consideration	 <br>Please harmonize the statements and explain more regarding this.<br><br><br><br><br>Explain the full meaning what is a unit (CG or IG).example CG=Control Group for the first of the statement.<br><br><br><br><br>Please add the reference number or approval code.
4	7,142	At the end of the trial, the HCPs from the two units (CG and IG) involved in the study were invited to participate in two focus groups, one for the CG and one for the IG.	I would like to suggest to the author provide more information on the methodology of the study such as:<br>-How about<br>the sample size selected?<br>-The total number of respondents?<br>-The inclusion and exclusion criteria?<br>-The interview protocol process consisted of what?

		Focus Group Discussion (FGD)	To briefly explain the interview protocol for FGD. What were the questions asked in this phase?
5	8,186-<br><br>17,383	Participants' comment	All the participant's statements must be italic
6	8,171	Those themes were regrouped into two dimensions: outcomes of the interventions and implementation in nursing care (see Figure).	No figure
7		References	10/39 of the references are more than 10 years. Recommendation: 5 years above is better.
