# Supplementary material for: Is massage a legitimate part of nursing care? A qualitative study
Source: PLoS One. 2023 Feb 27;18(2):e0281078. doi: 10.1371/journal.pone.0281078 (PMC9970055; doi:10.1371/journal.pone.0281078)
Supplement: Attachment — Submitted filename: Response_to_reviewers_27.10.22.docx [file pone.0281078.s002.docx]

**Response to Reviewers**

**Reviewer #1:**

Congratulation on the submitted manuscript. The topic is timely and will be of interest to the readers of the journal. However, few changes are suggested to improve the clarity of this manuscript. I have several recommendations and questions about the manuscript.

**Comment**

**Abstract**

**1. A qualitative study approach was used for this study involving how many participants? It is necessary to provide the total number of participants in your approach at the beginning of your statement.**

We have now clearly stated the numbers of participants in the focus groups that are at the core of this qualitative study (p. 2, para 48)

*“10 caregivers from the TM group and 6 from the machine group”*

**-The inclusion criteria for the study need to be discussed here.**

HCPs were included in this study if they participated in the training about TM or the use of the machine delivering massage. Thus, we added it in the text accordingly (p. 2, para. 46).

*“At the end of the trial, two focus groups were conducted with HCPs from each unit who took part in the training and agreed to discuss their experience: 10 caregivers from the TM group and 6 from the machine group.”*

**-List the Five themes that emerged from the thematic analysis in the abstract.**

This has been amended in the text accordingly (p. 2, para. 52)

*“Five themes emerged from thematic content analysis: perceived impact on patients, HCPs’ affective and cognitive experiences, patient-professionals relationships, organizational tensions, and conceptual tensions.”*

**Introduction**

**2. Need to explain the full meaning of The ICD-11 for the first use in a sentence.**

Indeed, it is important to specify it. Therefore, we added the full name this abbreviation (p. 4, para. 69)

*“The International Classification of Diseases 11th Revision (ICD-11)”*

**Methodology**

**3. An intervention (TM or control) was assigned to each of the two units/clusters (page 6,para 134). Please harmonize the statements and explain more regarding this.**

We thank the reviewer for his/her comment which allows us to improve the overall clarity of our setting. Two units of a general rehabilitation ward from a university hospital in Switzerland were selected for this study. We compared TM with a machine-delivered massage, using a non-randomized cluster clinical trial design. Each of these two interventions were assigned to one of the units. For better clarity, we modified the text accordingly (p. 6, para. 132).

*“The study was designed as a non-randomized cluster clinical trial with an exploratory qualitative part [32]. Two units of a general rehabilitation ward were selected for this trial. TM has been assigned to one unit (intervention group; IG) whereas machine-delivered massage has been assigned to the other (control group; CG).”*

**4. “The HCPs received training on TM or the use of the machine according to their unit (CG or IG)” (page 6, para 137). Explain the full meaning what is a unit (CG or IG). Example CG=Control Group for the first of the statement.**

Please see previous comment (3) for a better description of what unit (CG or IG) means.

**5. Ethical consideration- Please add the reference number or approval code.**

This has been amended in the text as advised (p. 7, para. 139).

*“This study received the approval of the Cantonal Commission for Ethics and Human Research in Geneva (CCER 2019-00848) and was pre-registered (ClinicalTrials.gov, NCT04295603) [32].”*

**6. I would like to suggest to the author provide more information on the methodology of the study such as:**

**-How about the sample size selected?**

We have now tried to be more explicit regarding the selection of the participants to the 2 focus groups and the total number of respondents. We added in the text as advised (p. 7, para. 150).

*“From the 21 HCPs working in the IG unit, 15 HCPs (7 nurses and 8 nursing auxiliaries) took part in the TM training and in the trial. In the CG unit all 21 HCPs working in this unit were offered participation, and 8 of them took part in the machine-delivered massage training and in the trial”*

**-The inclusion and exclusion criteria?**

Inclusion criteria were: to be working in one of the two units selected for this study and to have taken part to the training on either TM or the use of the machine. We modified the text accordingly (p. 7, para. 148)

*“HCPs were included if they were working in one of the two selected units and participated in the trainings whether or not they delivered the interventions. We aimed to include as many opinions as possible as our goal was to investigate the feasibility of the interventions.”*

**-The interview protocol process consisted of what?**

We have now added a paragraph regarding the choice of our investigation method (p. 7, para. 159)

*“Focus groups have been chosen to explore the satisfaction and general perception of massage in the multidisciplinary health care teams in the two units concerned. The choice of this method was of relevance in the context of the various units of the ward that all function as specific teams. Thus, a method of data collection that simultaneously generates data for three levels of analysis: the individual, the group and the interactions between participants was of clear interest. The protocol insisted on the need to recruit at least five participants, including nurses, assistant nurses, physical therapists and/or physicians. Based on the experience of our research team regarding TM, an interview guide was devised to assess the impact of massage on general care, the experience thereof, the positive and negative effects, and its impact on the development and planning of care (see S1 Table). The interview has been audio-recorded, and transcribed verbatim.”*

**- To briefly explain the interview protocol for focus group discussion (FGD). What were the questions asked in this phase?**

We thank the reviewer for his/her suggestions. Therefore, we added a brief description of the dimensions explored in the interview guide and give examples of questions (p. 8, para. 170)

*“The interview guide explored the HCPs’ experiences and use of the interventions (see Table 1). Four dimensions were investigated : recall of the massage, general appreciation of massage, facilitators and barriers in the experience of massage, and benefits for other.”*

**7. All the participant’s statements must be italic**

This has been amended in the manuscript as advised.

**8. “Those themes were regrouped into two dimensions: outcomes of the interventions and implementation in nursing care (see Figure)”. No figure attached**

Indeed, following the submission guidelines of Plos One, Figures should not be included in the manuscript but should be uploaded as a separate document. Thus, we included only the figure caption in the manuscript. For your information, figure 1 is attached below:

**9. References: 10/39 of the references are more than 10 years. Recommendation: 5 years above is better.**

We agree with the reviewer. However, some of the articles are at the foundation of a theory (e.g. Braun and Clarke, 2006 regarding thematic analysis; or Rogers, 1970, and Newman, 2008, regarding the theoretical basis of nursing), or are regarded as landmarks in their field (e.g. Malterud, 2001 challenges and guidelines for the use of qualitative methods outside of social sciences; or Kitzinger, 1994 which still provides a very well-informed methodology for focus groups) or we just could not find more recent article. We carefully checked that our “old” articles could not be replaced by more recent ones.
